# Application of Bidirectional Generative Adversarial Networks to Predict Potential miRNAs Associated With Diseases

**DOI:** 10.3389/fgene.2022.936823

**Published:** 2022-07-12

**Authors:** Long Xu, Xiaokun Li, Qiang Yang, Long Tan, Qingyuan Liu, Yong Liu

**Affiliations:** ^1^ School of Computer Science and Technology, Heilongjiang University, Harbin, China; ^2^ Postdoctoral Program of Heilongjiang Hengxun Technology Co., Ltd., Heilongjiang University, Harbin, China; ^3^ School of Electronic Engineering, Heilongjiang University, Harbin, China

**Keywords:** microRNAs, complex disease, MiRNA–disease association prediction, similarity network, bidirectional generative adversarial network

## Abstract

Substantial evidence has shown that microRNAs are crucial for biological processes within complex human diseases. Identifying the association of miRNA–disease pairs will contribute to accelerating the discovery of potential biomarkers and pathogenesis. Researchers began to focus on constructing computational models to facilitate the progress of disease pathology and clinical medicine by identifying the potential disease-related miRNAs. However, most existing computational methods are expensive, and their use is limited to unobserved relationships for unknown miRNAs (diseases) without association information. In this manuscript, we proposed a creatively semi-supervised model named bidirectional generative adversarial network for miRNA-disease association prediction (BGANMDA). First, we constructed a microRNA similarity network, a disease similarity network, and Gaussian interaction profile kernel similarity based on the known miRNA–disease association and comprehensive similarity of miRNAs (diseases). Next, an integrated similarity feature network with the full underlying relationships of miRNA–disease pairwise was obtained. Then, the similarity feature network was fed into the BGANMDA model to learn advanced traits in latent space. Finally, we ranked an association score list and predicted the associations between miRNA and disease. In our experiment, a five-fold cross validation was applied to estimate BGANMDA’s performance, and an area under the curve (AUC) of 0.9319 and a standard deviation of 0.00021 were obtained. At the same time, in the global and local leave-one-out cross validation (LOOCV), the AUC value and standard deviation of BGANMDA were 0.9116 ± 0.0025 and 0.8928 ± 0.0022, respectively. Furthermore, BGANMDA was employed in three different case studies to validate its prediction capability and accuracy. The experimental results of the case studies showed that 46, 46, and 48 of the top 50 prediction lists had been identified in previous studies.

## 1 Introduction

MicroRNAs (miRNAs) are endogenous gene-encoded non-coding single-stranded RNA molecules with a length of about 20–24 nucleotides (Bartel, 2004; Cheng et al., 2005). They participated in the post-transcriptional management of gene expression and are crucial for multiple biochemical processes (such as cellular apoptosis, cell proliferation, metabolism, and so on) by targeting specific message RNAs (mRNAs) and regulating gene expression and miRNA degradation (Ambros, 2003; Karp and Ambros, 2005; Shukla et al., 2011; Ebert and Sharp, 2012; Shi et al., 2013). Therefore, scientists are increasingly interested in human biochemical processes at the miRNA level (Xu et al., 2004). Currently, accumulating evidence indicates that most miRNAs are directly related to complex human diseases (Meltzer, 2005; Krützfeldt and Stoffel, 2006; Jiang et al., 2009; Chen et al., 2019). For instance, it was confirmed that mir-367 can facilitate the proliferation of hepatocellular carcinoma and invasion of cells, displaying aberrant expressions in the tumor tissues of hepatocellular cancer patients compared to normal tissues (Guo et al., 2020). In addition, recent studies revealed that mir-21 is correlated with the prognosis of patients with brain tumors, and particularly, its overexpression may cause a worse prognosis (He et al., 2016). Another example of disease-related miRNA is mir-125a-5p, which plays a critical role in lung cancer development under the regulation of epidermal growth factor signal transduction (Wang et al., 2009). Therefore, utilizing experimental or computational models to identify the underlying miRNA–disease associations will contribute to facilitating the discovery of pathogenic mechanisms and potential biomarkers (Calin and Croce, 2006; Jones et al., 2014). Previous studies have indicated that predicting possible miRNA–disease associations through traditionally biological methods is laborious and expensive. With the rapid evolution of technology and science, numerous advanced computational methods have been developed to establish the representation of pairwise miRNA-disease associations based on accumulated genomic data.

Based on the assumption that functionally similar mirnas are likely to be associated with phenotypically similar diseases (Perez-Iratxeta et al., 2005), researchers had made great progress over the past few decades in building computational models to infer potential miRNA-disease associations. Most of them were score function-based models, which analyze biological information to establish the score function and predict the associations (Zeng et al., 2016b). For example, an innovative model named MiRNA-protein-disease Association was proposed (Mørk et al., 2014), which utilized proteins as mediums between miRNAs and diseases to predict the associations. A group of proteins correlated with a specific miRNA (disease) was listed, the association scores of miRNA-protein pairs and protein-disease pairs were calculated, and the maximum value was selected as the final association score is between miRNA and disease. In addition Chen et al. (2016) mixed the known miRNA-disease associations, the diseases semantic similarity, and the miRNAs functional similarity with GIP kernel similarity to figure out unknown miRNA-disease associations. Then, a model named within and between score for MiRNA-disease association prediction (WBSMDA) was developed, which can simultaneously prioritize miRNAs for all diseases. A new similarity matrix of miRNA–disease associations was constructed (Ma et al., 2019) by integrating gene similarity information, miRNA target gene information, disease gene information, and other data sources. They applied the nuclear neighborhood similarity algorithm to calculate the similarity feature of miRNA-disease pairs. Ultimately, a bidirectional propagation algorithm was adopted to obtain the predicted score.

In the past 2 decades, machine learning-based models have been widely proposed to predict the underlying associations of miRNA-disease pairs. For example, researchers constructed a vector spacer model named MiRAI (Pasquier and Gardè s, 2016). First, an adjacent matrix was acquired by splicing four types of miRNA-related associations among disease, target, neighbor, and cluster. Then, they applied a singular value decomposition algorithm to cut down the new matrix dimension and computed an eigenvector representation of each miRNA–disease pair. Finally, the correlation grade was obtained using the cosine similarity of miRNA–disease vector representation. Based on combining two ideal classifiers in disease (miRNA) space to optimize the association probability, a model named Regularized Least Squares for predicting miRNA-disease associations (RLSMDA) was proposed (Chen and Yan, 2014). It is worth mentioning that this method does not require any representation of unknown miRNA–disease pairs. An inductive matrix completion model was developed (Chen et al., 2018) to predict the disease-related miRNAs, which is applicable to new diseases with unknown miRNAs. First, they set up two metrics to indicate the low-dimensional matrix of miRNA-disease representation. Then, an optimal algorithm was used in iteration to update them. When the stopping threshold was reached, the two updated matrices were directly fused into the miRNA-disease similarity matrix. As an enhancement, researchers innovatively constructed an updated model named Neighborhood Constraint Matrix Completion for miRNA-disease association prediction (NCMCMDA), which utilized the similarity information of miRNAs and diseases (Chen *et al.* (2021)). They applied a fast iterative shrinkage-thresholding algorithm based on the known miRNA-disease associations and comprehensive miRNA (disease) similarity to recover the missing association information. A label propagation-based method was proposed (Li et al., 2018) for scoring miRNA-disease pairs by calculating pairwise neighborhood similarity (LPLNS). Due to unvalidated miRNA-disease pairs presenting few known associations, an additional processing step was included in the LPLNS to drive new interaction likelihood profiles. To list the candidate miRNAs for diseases and explore the potential associations, a novel framework called GBDT-LR was constructed by combining logistic regression and gradient boosting decision tree (Zhou et al., 2020). Besides, researchers developed a computational model based on Similarity Constrained Matrix Factorization for miRNA-disease association prediction (Li et al., 2021a), which creatively expanded L2 regular term and similarity constraint term to infer disease-related miRNAs.

Many novel neural network-based methods that extract similarity features and learn the latent representations had been proposed to predict potential miRNA-disease associations (Yang and Li, 2021). For example, a model called Deep Belief Network for miRNA-disease association prediction (DBNMDA) was mentioned (Chen et al., 2021), which innovatively constructed the feature vectors with all microRNA and disease information to pre-train restricted Boltzmann machines. A graph neural network-based auto-encoder model called GAEMDA (Li et al., 2021b) adopted an end-to-end way to identify the underlying associations between miRNAs and diseases. By combining an auto-encoder and a convolutional neural network (Peng et al., 2019), a learning-based neural network model was constructed to figure out the potential miRNA-disease associations. Based on four integrated biological networks and verified protein-protein interaction in humans, researchers developed a new computational framework named Heterogeneous Graph Convolutional Network for miRNA-disease associations prediction (Li et al., 2019). For predicting the correlations between miRNAs and diseases, some studies presented a supervised end-to-end method, termed the neural inductive matrix completion with graph convolutional network (Li et al., 2020), which can effectively learn the representation of underlying traits from the known miRNA (disease) information. To learn the original and global miRNA-disease representations in a low-dimensional feature space (Xuan et al., 2019), a novel network-based model was proposed, termed Convolutional Neural network for miRNA-disease associations prediction (CNNMDA).

Despite the great progress achieved in the techniques used to explore the potential miRNA and disease relationships, the above-mentioned models, and also others, still present some restrictions and disadvantages. Overall, the studies published in the past decade show that great success has been achieved in the field of bioinformatics based on deep learning-based models. In particular, computational models based on neural networks have made outstanding contributions to the task of prediction (Zeng et al., 2016a). As a neural network, the auto-encoder can learn input data through unsupervised learning, strongly represent potential features, effectively reduce sample noise, and randomly generate data (Suk et al., 2015). For example, aiming to discover complex feature representation of disease-related miRNAs, a novel deep learning method for predicting miRNA-disease associations through deep autoencoder with multiple kernel learning (DAEMKL) was presented (Zhou et al., 2021). In this study, a creative computational model named bidirectional generative adversarial network (BGANMDA) is proposed to predict potential pairwise miRNA-disease associations. More specifically, similarity information networks were initially constructed from comprehensive similarity characteristics. Then, a similarity feature network with the full underlying relationships between miRNA and disease pairs was obtained by integrating all the similarity information. The whole integrated similarity network was loaded into the BGANMDA which employed an encoder to learn high-level features in latent space, a generator to produce a brand-new correlation between miRNAs and diseases, and a discriminator to decide whether the predicted associations were real. In this study, five-fold cross-validation and leave-one-out cross-validation (LOOCV) were adopted to evaluate the model’s prediction performance. During the five-fold cross-validation, the BGANMDA acquired an area under the curve (AUC) of 0.9319 and a standard deviation of 0.00021 was obtained. At the same time, in the global and local leave-one-out cross-validation (LOOCV), the AUC value and standard deviation of BGANMDA were 0.9116 ± 0.0025 and 0.8928 ± 0.0022, respectively. Furthermore, BGANMDA was employed in three different case studies to validate its prediction capability and accuracy. The experimental results of the case studies showed that 46, 46, and 48 of the top 50 prediction lists had been identified in previous studies.

## 2 Materials and Methods

### 2.1 Human miRNA–Disease Associations

The Human MiRNA Disease Database (HMDD V3.0) was adopted as a benchmark dataset (Huang et al., 2019), which can be directly downloaded for experimental verification of disease-related miRNA information from http://www.cuilab.cn/hmdd (version v 3.2, published on 27 Mar 2019). After erasing the pairwise miRNA-disease associations that did not have IDs or lacked traits, duplicate samples describing the miRNA-disease relationships were removed based on experimental support. In the process, 18,733 miRNA-disease associations were obtained, including 1,208 miRNAs and 985 diseases in the HMDD v 3.2 database. Based on the sorted dataset, we constructed an association binary matrix *BM*, consisting of 984 rows and 1,207 columns, was constructed to maintain the interaction information between miRNAs and diseases, which has 984 rows and 1,207 columns. If an experimentally verified miRNA-disease association was detected, the element value at the corresponding position of the matrix was set to 1; otherwise, it was set to 0.

### 2.2 Multi-Source Similarity Information for miRNAs and Diseases

#### 2.2.1 MiRNA Similarity Network

**FIGURE 1:**
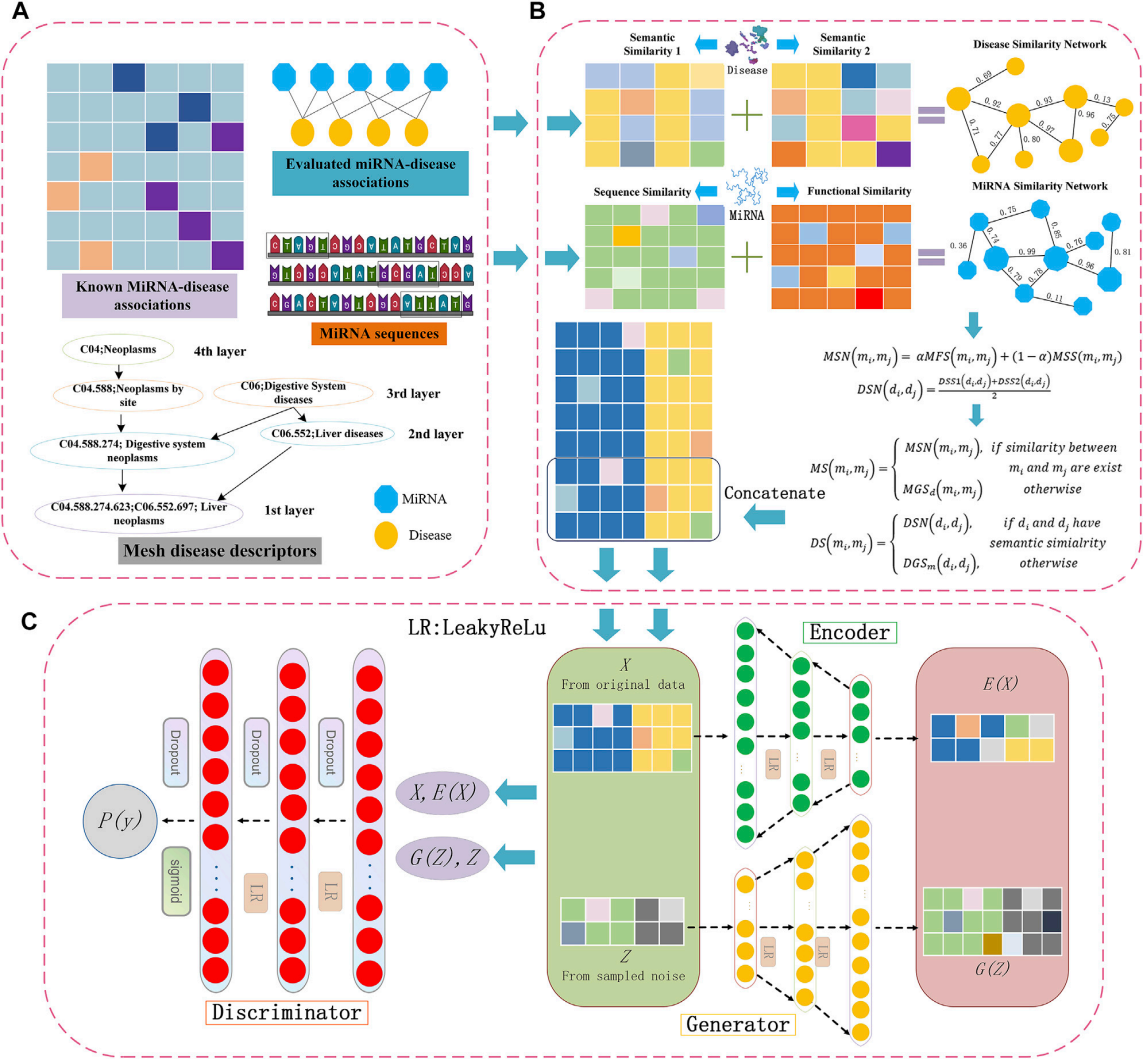
Flowchart of potential miRNA-disease association prediction based on the BGANMDA model, **(A)** is the multi-source information of miRNAs and diseases, **(B)** is the details of constructing both miRNAs and diseases similarity network, and **(C)** is the structure of BGANMDA.

To calculate the network of miRNA sequence similarity, the miRBase database containing almost all the miRNA sequence information, (Kozomara and Griffiths-Jones, 2014), was downloaded from https://www.mirbase.org, as shown in Figure 1A. The similarities of any two miRNAs were quantified using the Levenshtein distance, which represented the minimum cost of converting a single string to another string after replacing, inserting, and deleting one letter. The editing penalty was set to 2, while the deleted and inserted penalties were set to 1. Let *MSS*(*m*_*i*_, *m*_*j*_) be the miRNA similarity score, where *m*_*i*_ denotes the *ith* miRNA and *m*_*j*_ denotes the *jth* miRNA, and the definition formula is shown as follows:

$$MSS\left(m_{i},m_{j}\right)=1-\frac{x}{Len\left(m_{i}\right)-Len\left(m_{j}\right)},$$ (1)

where *x* denotes the minimum penalty and *Len*(*m*) is the sequence length of miRNA.

Then, we applied the max-min normalization to rescale and normalize *MSS* as follows:

$$MSS^{\prime }=\frac{MSS\left(m_{i},m_{j}\right)-MSS_{min}}{MSS_{max}-MSS_{min}},$$ (2)

where *MSS*_max_ and *MSS*_min_ represent the maximum and minimum *MSS*, respectively.

Based on the universally acknowledged conjecture that pathologically similar diseases are more likely to be relevant to functionally similar miRNAs (and vice versa), a popular strategy (Zou et al., 2016) was employed to calculate the functional similarity (*MFS*) for a miRNA-miRNA pair *m*_*i*_ and *m*_*j*_ as follows:

$$MFS=\frac{\sum_{d\in D\left(m_{i}\right)⋂\;D\left(m_{j}\right)}\left(DSS\left(d,\;D\left(m_{i}\right)\right)\;+\;DSS\left(d,\;D\left(m_{j}\right)\right)\right.}{\left|D\left(m_{i}\right)|+|D\left(m_{j}\right)\right|},$$ (3)

where *D* (*m*_*i*_) and *D* (*m*_*j*_) denote the disease sets linked with *m*_*i*_ and *m*_*j*_, respectively, and |*D* (⋅)| is the cardinal number of disease set. *DSS*(*d*, *D* (*m*_*j*_)) is calculated through the max value of disease semantic similarity.

In this study, the sequence and functional similarity of miRNAs were employed to describe the miRNA similarity characteristics. However, an independent similarity descriptor is not capable of accurately expressing the global similarity between any two miRNAs. Therefore, a similarity factor was set to determine which similarity descriptor can better represent similar features of miRNAs. Finally, a miRNA similarity network *MSN*(*m*_*i*_, *m*_*j*_) between miRNA *m*_*i*_ and *m*_*j*_ was obtained by synthesizing the two similarity descriptors as follows:

$$MSN\left(m_{i},m_{j}\right)=\alpha MFS\left(m_{i},m_{j}\right)+\left(1-\alpha \right)MSS\left(m_{i},m_{j}\right),$$ (4)

where *α* is the similarity factor. In order to better describe the similarity characteristics between mirnas, we carefully compared the differences in miRNA functional similarity and sequence similarity in the description of similarity characteristics. Referring to previous literature (Lei et al., 2021), it was found that mirnas with similar functions were more likely to cause similar diseases. Experimental comparison shows that the optimal miRNA similarity network can be constructed when the value of *α* is set to 0.6.

#### 2.2.2 MiRNA GIP Kernel Similarity

Based on the previous study, the Gaussian interaction profile can be applied to capture the topological features of interaction networks, which is a successful method to evaluate the nuclear similarity of biomolecules for each pair in computational biology (Chen et al., 2016; Bao et al., 2017). Therefore, it is of great importance to search for GIP kernel similarity in the binary matrix with miRNA-disease interaction information. The GIP kernel similarity of microRNA *MGS*_*d*_ (*m*_*i*_, *m*_*j*_) between *m*_*i*_ and *m*_*j*_ can be defined as follows:

$$MGS_{d}\left(m_{i},m_{j}\right)=exp\left(-\lambda \|BM\left(m_{i}\right),BM\left(m_{j}\right){\|}^{2}\right),$$ (5)

where *BM*(*m*_*i*_) and *BM*(*m*_*j*_) are the *ith* and *jth* columns miRNA kernel information of each disease in binary matrix *BM*, respeectively, *λ* represents a parameter that controls the kernel boundary’s width, which can be calculated based on *λ*′ as follows:

$$\lambda =\frac{\lambda^{\prime }}{\frac{1}{nm}\sum_{i=1}^{nm}\|BM\left(m_{i}\right)\|},$$ (6)

where *nm* denotes the number of miRNAs, *λ*′ is the original bandwidth and set to 1 as suggested in other studies (Xiao et al., 2019).

#### 2.2.3 Disease Similarity Network

As described in previous studies (Xuan et al., 2013), by using the medical subject headings disease descriptors (MeSH), downloaded from the NCBI website (https://www.ncbi.nlm.nih.gov/), it was possible to estimate the semantic similarity of diseases based on directed acyclic graph (DAG) structures. Specifically, a disease *d* can be described as *DAG*_*d*_ = (*d*, *G*(*d*), *E*(*d*)), in which *G*(*d*) denotes the disease *d* itself and all the node of its ancestors, *E*(*d*) is the corresponding edge set that contains the direct relationships from parents to child nodes in DAGs. Therefore, we computed the semantic contribution of disease *d*_*k*_ and *d*_*t*_ as follows:

$$C1_{d_{t}}\left(d_{k}\right)=\left\{\begin{array}{c} max\left\{\mu \cdot C1_{d_{t}}\left(d_{k}^{\prime }\right)\right\},\;if\;d_{k}\neq d_{t}\;\\ \;\;1,\;\;\;if\;d_{k}=d_{t}\; \end{array}\right.,$$ (7)

where *μ* is the semantic contribution factor, as suggested by recent study we set it as 0.5 (Wang et al., 2010). Then, the disease semantic value *d*_*t*_ is defined as

$$SV1\left(d_{t}\right)=\underset{d_{k}\in G_{\left(d\right)}}{\sum }C1_{d_{t}}\left(d_{k}\right).$$ (8)

Let *DSS*1 ∈ *R* ^*nd*×*nd*^ be the pairwise disease semantic similarity, which can be computed as follows:

$$DSS1\left(d_{i},d_{j}\right)=\frac{\sum_{x\in G\left(d_{i}\right)\cap G\left(d_{j}\right)}\left(C1_{d_{i}}\left(x\right)+C1_{d_{j}}\left(x\right)\right)}{SV1\left(d_{i}\right)+SV1\left(d_{j}\right)},$$ (9)

where $G_{\left(d_{i}\right)}$ and $G_{\left(d_{j}\right)}$ represent the disease *d*_*i*_ and *d*_*j*_ in DAGs, respectively, and *DSS*1 is the first matrix to store the disease semantic similarity.

Furthermore, because diseases are more common when they appear in more DAGs, they are more specific when they appear in fewer GAGs, and in the same DAG layer, the diseases' semantic contribution value is almost different. Therefore, based on previous research (Pasquier and Gardès, 2016), another measurement was applied to obtain the semantic similarity of diseases as follows:

$$C2_{d_{t}}\left(d_{k}\right)=-log\left(\frac{NG\left(d_{k}\right)}{nd}\right),$$ (10)

where *NG* (*d*_*k*_) is the number of DAGs including *d*_*k*_.

Similarly, both the disease semantic value *d*_*t*_ and the pairwise disease semantic similarity *DSS*2(*d*_*i*_, *d*_*j*_) can be described as follows:

$$SV2\left(d_{t}\right)=\underset{d_{k}\in G_{\left(d\right)}}{\sum }C2_{d_{t}\left(d_{k}\right)}$$ (11)

$$DSS2\left(d_{i},d_{j}\right)=\frac{\sum_{x\in G\left(d_{i}\right)\cap G\left(d_{j}\right)}\left(C2_{d_{i}}\left(x\right)+C2_{d_{j}}\left(x\right)\right)}{SV2\left(d_{i}\right)+SV2\left(d_{j}\right)},$$ (12)

where *DSS*2 is the second matrix to store the semantic similarity of diseases.

To obtain a more persuasive semantic similarity of diseases, a disease similarity network *DSN*(*m*_*i*_, *m*_*j*_) was constructed between disease *d*_*i*_ and *d*_*j*_ by coalescing the two semantic disease similarities as follows:

$$DSN\left(d_{i},d_{j}\right)=\frac{DSS1\left(d_{i},d_{j}\right)+DSS2\left(d_{i},d_{j}\right)}{2}.$$ (13)

#### 2.2.4 Disease GIP Kernel Similarity

Correspondingly, the GIP kernel similarity of diseases can be established as follows:

$$DGS_{m}\left(d_{i},d_{j}\right)=exp\left(-\lambda \|BM\left(d_{i}\right),BM\left(d_{j}\right){\|}^{2}\right)$$ (14)

$$\lambda =\frac{\lambda^{\prime }}{\frac{1}{nd}\sum_{i=1}^{nd}\|BM\left(d_{i}\right)\|},$$ (15)

where *BM*(*d*_*i*_) and *BM*(*d*_*j*_) represent the *ith* and *jth* rows disease kernel information of each miRNA in binary matrix BM, respectively, *λ* represents a parameter that controls the width of the kernel boundary, and *nd* is the number of diseases.

### 2.4 Integrated Similarity Characteristic

Based on the above section, the miRNAs (diseases) similarity network and the GIP kernel similarity of miRNAs (diseases) were collected to acquire comprehensive similarity information. Considering that many sparse values may exist in the above-mentioned similarity network, the GIP kernel similarity was integrated with the similarity networks of miRNAs and diseases using the following formulas:

$$MS\left(m_{i},m_{j}\right)=\left\{\begin{array}{c} MSN\left(m_{i},m_{j}\right),\;if\;similarity\;between\;\\ \;\;\;\;m_{i}\;and\;m_{j}\;are\;exist\;\\ MGS_{d}\left(m_{i},m_{j}\right),\;\;otherwise\; \end{array}\right.,$$ (16)

$$DS\left(d_{i},d_{j}\right)=\left\{\begin{array}{c} DSN\left(d_{i},d_{j}\right),\;if\;d_{i}\;and\;d_{j}\;have\;\\ \;\;\;\;semantic\;similarity\;\\ DGS_{m}\left(d_{i},d_{j}\right),\;\;otherwise\; \end{array}\right..$$ (17)

In the integrated similarity network, the miRNA similarity vector of miRNA *m*_*i*_ stores the similarity values of all the miRNAs to *m*_*i*_. At the same time, the similarity values of all other diseases to disease *d*_*i*_ are included in the similarity vector of *d*_*i*_. Thus, all the similarity eigenvectors for the corresponding pairwise miRNA-disease were concatenated to create a long feature vector of size *nm* + *nd*, where the *nm* and *nd* represent the number of microRNAs and diseases, respectively. Overall, the *nm* × *nd* generated eigenvectors were taken as samples, each sample corresponding to a miRNA-disease pair. As shown in Figure 1B, the details of processing similarity for potential miRNA-disease prediction is displayed.

### 2.5 Bidirectional Generative Adversarial Network

The present study introduced a computational model named bidirectional generative adversarial network (BGANMDA), which combined a nonlinear auto-encoder (consisting of an encoder and a generator), and an optimal discriminator to complete the task of identifying the potential associations between miRNAs and diseases. The Framework of BGANMDA is shown in Figure 1C (See more details in Supplementary Figure S2). Generally, the encoder of BGANMDA maps the original data point *x* to the feature representation *E*(*x*) in latent space. At the same time, *z* is captured to generate a new relationship between a miRNA and a disease *G*(*z*) through hidden layers in the generator. Then, the BGANMDA discriminator discriminates both in the traditional data space, and in the joint data and latent space ((*x*, *E*(*x*)) versus (*G*(*z*), *z*)), where the output of encoder *E*(*x*) and the input of generator z are the latent components.

In the model, the encoder *E*: Ω_*X*_ → Ω_*z*_ included a distribution *P*_*E*_ (*Z*|*X*) = *δ*(*Z* − *E*(*x*)), which maps the original data points *x* into a latent space of the generator. At the same time, the generator *G*: Ω_*Z*_ → *Omega*_*X*_ randomly extracts sampling noise form the latent space of the encoder to generate new miRNA-disease associations under the distribution *Q*_*G*_ (*X*|*Z*) = *δ*(*X* − *G*(*z*)). To “fool” a discriminator perfectly, both of encoder and generator must learn to invert each other through the joint probability distribution, satisfying the following two properties:

$$\left(a\right)X\in {\hat{\Omega }}_{X}\wedge E\left(X\right)=Z\;\left(b\right)Z\in {\hat{\Omega }}_{Z}\wedge G\left(Z\right)=X.$$ (18)

The discriminator will take (*X*, *Z*) as input from the latent space to forecast the deterministic relationship of miRNA-disease pairs under the distribution of *P*_*D*_ (*Y*|*X*, *Z*). If only property (a) is satisfied, the discriminator can infer the source of input (*X*, *Z*) must be come from the encoder pair (*X*, *E*(*X*)), and the value of discriminator $D_{EG}^{*}\left(X,Z\right)$ is 1; if the source of (*X*, *Z*) only satisfies (b), it must be come from the generator pair (*G*(*Z*), *Z*) and $D_{EG}^{*}\left(X,Z\right)$ is 0. Therefore, a minimax objective can be defined to displace the BGANMDA training objective as follows:

$$\underset{G,E}{min}\underset{D}{max}V\left(D,E,G\right),$$ (19)

where *V* (*D*, *E*, *G*) can be represented by the following formulas:

$$E_{X∼p_{X}}\left[logD\left(X,E\left(X\right)\right)\right]+E_{Z∼p_{Z}}\left[log\left(1-D\left(G\left(Z\right),Z\right)\right)\right]$$ (20)

and

$$logD\left(X,E\left(X\right)\right)=E_{Z∼p_{E}\left(\cdot |X\right)}\left[logD\left(X,Z\right)\right],$$ (21)

$$log\left(1-D\left(G\left(Z\right),Z\right)\right)=E_{X∼p_{G}\left(\cdot |Z\right)}\left[log\left(1-D\left(X,Z\right)\right)\right].$$ (22)

When a discriminator input (*X*, *Z*) satisfies noth (a) and (b), *E* and *G* invert each other almost everywhere, that is *X* = *G* (*E*(*X*)) and *Z* = *E* (*G*(*Z*)). Compared with other advanced miRNA-disease predicting models, the BGANMDA here employed focuses more on processing complex data and effectively learning the gradient information to ensure the correct allocation of parameter weights.

In preprocessed similarity eigenvectors, each disease (miRNA) contains the similarity information of all miRNAs (diseases), which integrate the miRNAs similarity network, diseases similarity network, and GIP kernel similarity of miRNAs (diseases). As mentioned above, BGANMDA is an innovative computational model inspired by a nonlinear auto-encoder. A BGANMDA encoder is one of the two parts of an auto-encoder, and it shows a strong performance in terms of compressing complex data, eliminating extra noise, and learning the additional features of latent space. The eigenvector samples are used as encoder input and the parameters of the similarity vectors can be calculated through three fully connected layers of the neural network. Besides, LeakyReLu was applied as an activation function for each network layer with dropout to accelerate the convergence rate and prevent the occurrence of over-fitting. It assigns a non-zero slope to all negative values, which can accelerate gradient descent and better carry out backpropagation The function is defined as follows:

$$LeakyReLu=\left\{\begin{array}{c} x,\;\;x\geq 0\;\\ \eta x,\;x<0\; \end{array}\right.,$$ (23)

where *η*, which is set to 0.01, denotes a fixed learning parameter.

In the BGANMDA encoder, the dimensions of the similarity eigenvector samples between miRNA and disease are compressed into low-dimensional vectors when passing through network layers. The dense information of compressed low-dimensional vectors allows the model to learn how to map the miRNA-disease relationship into latent space. In this way, a trained encoder can precisely identify the feature representation by capturing semantic attributes, in order to obtain a data pair (*x*, *E*(*x*)). To better understand the further representation of latent space, the number of neurons in an encoder output layer was set to 100. At the same time, the binary cross-entropy was used as the loss function, as shown in the following equation:

$$Loss_{p}\left(q\right)=-\frac{1}{\Gamma }\overset{\Gamma }{\underset{i=1}{\sum }}y_{i}\cdot log\left(p\left(y_{i}\right)\right)+\left(1-y_{i}\right)\cdot log\left(1-p\left(y_{i}\right)\right),$$ (24)

where Γ represents the output size of BGANMDA, *y* is the label (1 for known miRNA-disease pairs), and *p*(*y*) is the predicted probability of the association between miRNA and disease.

In most generative adversarial network models, generators always play a role in studying the features of original data to generate new data based on the learned characteristics. However, in the BGANMDA, the generator was preferentially used to select a random sample as input instead of the original one. The structure of the generator network is similar to that of the encoder which has three fully connected layers with dropout. The generator output was calculated as follows:

$$G\left(Z\right)=W^{G}z+b^{G},$$ (25)

where *z* is the sampling noise from the encoder latent space, and *W* ^*G*^ and *b* ^*G*^ represent the weights and bias of the generator, respectively.

It is noteworthy that each layer in the BGANMDA generator increases the dimension of the potential representation layer by layer and the final output dimension is the same as the encoder input. The sampling noise representation of the miRNA-disease association is decoded by the generator, then the new associations are generated. As a result, a series of data pairs (*G*(*z*), *z*) is obtained.

The data pairs (x,E(x)) and (G(z),z) are taken as inputs to try to fool the discriminator. Initially, if the data pair derives from the encoder, the discriminator can easily recognize the input source and discriminate it as real, namely $D_{EG}^{*}\left(X,Z\right)$ is set to 1; whereas, if the data pair derives from the generator, the *D*_*E*_ *G*∗(*X*, *Z*) is set to 0. As the model comprehensively analyzes the underlying features of miRNA-disease relationships, the encoder and the generator learn to convert each other. It becomes difficult for the discriminator to distinguish the source of the input, so that we can obtain predictions that are more representative of reality. The sigmoid function was employed to calculate the final probability of miRNA and disease pairs, which is defined as follows:

$$sigmoid\left(\theta \right)=\frac{1}{1-exp\left(-\theta \right)},$$ (26)

where *θ* denotes the sigmoid function input.

This BGANMDA encoder, with its excellent representation ability, can learn the potential association of miRNA-disease pairs. The generator can extract the features from the sampled noise latent space and generate new miRNA-disease associations. Finally, *x* = *G* (*E*(*x*)) and *z* = *E* (*G*(*z*)) are almost everywhere through a union probability distribution to obtain a bidirectional structure. The experimental results of the present study reveal that BGANMDA is robust and has a strong representational learning ability to predict potential miRNA-disease associations and, compared with other state-of-the-art methods, it performs remarkably well.

## 3 Resluts

### 3.1 Performance Evaluation

In this study, we implemented BGANMDA based on the structure of combined generative adversarial networks with auto-encoder. The input size of BGANMDA encoder is 2,191, and the output size is 100. The generator in our model has the same network structure with encoder, its input size and output size are 100 and 2,191, respectively. The data pair (*x*, *E*(*x*)) or (*G*(*z*), *z*) was concatenated and fed to the BGANMDA discriminator, which has the dimension 2,291 of input size. We adopted Adam as a gradient descent algorithm to optimize parameters and the learning rate was fixed at 2e-4. To avoid over-fitting, the cross-entropy function and LeakyRuLe (Wang et al., 2018) were used as the loss function and activation function, respectively. The suitable dropout rate strategy ranged from 0.1 to 0.9, and we set it at 0.5 after the effective validation. To train the model, 20% of the evaluated samples in training sets were randomly removed five times, and the epochs and batch size were set at 20,000 and 128 each time.

**FIGURE 2:**
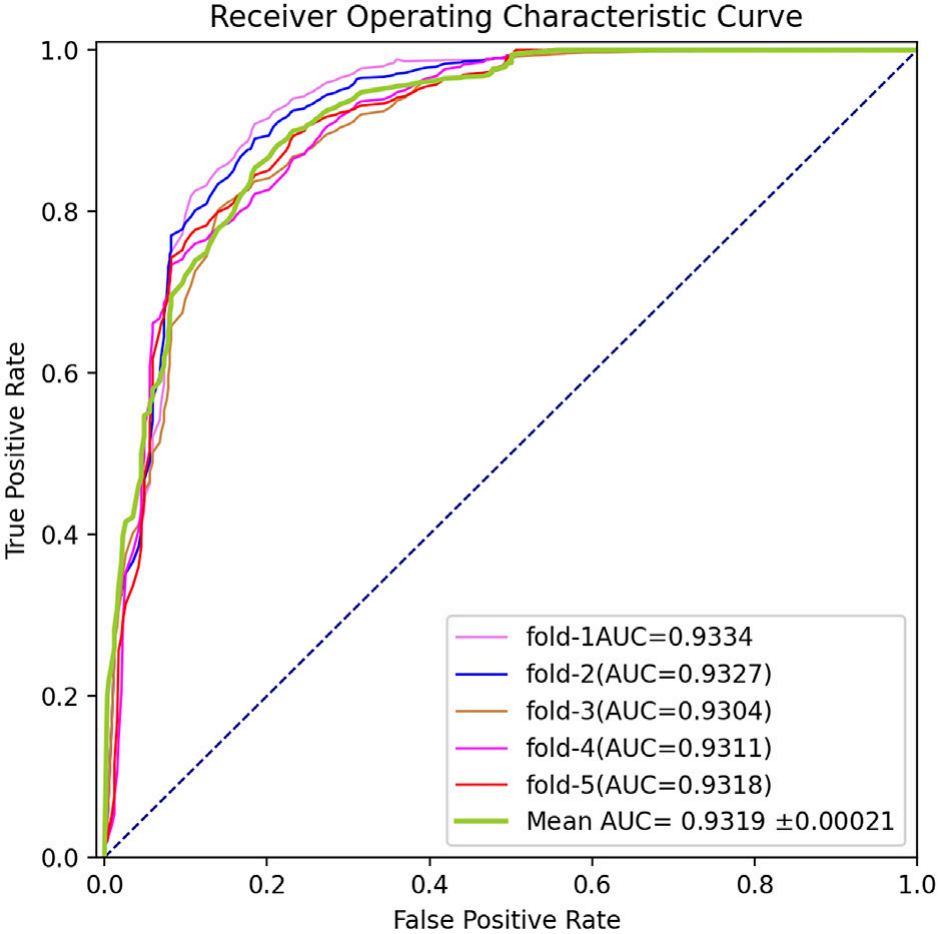
ROC curves performed in 5-flod cross-validation by BGANMDA, which obtained the mean AUC value and standard deviation of 0.9319 ± 0.0021.

To systematically measure the prediction capability of BGANMDA, three different validation methods were employed, namely five-fold cross-validation, global LOOCV, and local LOOCV. In the first, the acknowledged miRNA-disease samples were stochastically split into five subsets, where each was considered as the dataset for testing, and the others were treated as training sets. The BGANMDA model was used to prioritize the unverified miRNA-disease candidates and test samples according to the score given obtained. In order to ensure the reliability of the evaluation results, the five-fold cross-validation was repeated 150 times to reach the distribution of original samples. As shown in Figure 2, our model calculated a mean AUC and standard deviation of 0.9116 ± 0.00021. Meanwhile, we compared with other state-of-the-art models, namely the NCMCMDA, DBNMDA, GAEMDA, TDRC, SSCMDA, CNNMDA HDMP, KATZMDA, and WBSMDA via different evaluation index. As shown in Table 1, BGANMDA obtains the values of AUPR (0.9237), F1-score (0.9024), Recall (0.8968), Precision (0.9215), and MCC (0.8913), which outperforms than other advanced models in 5-fold cross validation. Noting that the miRNA (diseases) similarity network and GIP kernel similarity in 5-fold cross-validation process, because the correlation binary matrix was altered when part of the known association of miRNA-disease pairs was removed.

**TABLE 1:** The AUC AUPR, F1-scores, recall, precision, and MCC of ten methods on miRNA–disease associations prediction task in five-fold cross validation.

	AUC	AUPR	F1-score	Recall	Precision	MCC
BGANMDA	**0.9316**	**0.9237**	0.9024	0.8968	**0.9215**	**0.8913**
NCMCMDA	0.9187	0.9093	**0.9031**	0.8621	0.9033	0.8541
DBNMDA	0.8994	0.8972	0.8793	0.8517	0.8802	0.8632
GAEMDA	0.8674	0.8625	0.8609	0.8485	0.8756	0.8427
TDRCMDA	0.8713	0.8706	0.8569	**0.8990**	0.8783	0.8409
SSCMDA	0.8531	0.8654	0.8542	0.8501	0.8697	0.8371
CNNMDA	0.8494	0.8583	0.8512	0.8453	0.8724	0.8347
HDMP	0.8226	0.8407	0.8373	0.8486	0.8531	0.8287
KADZMDA	0.8317	0.8351	0.8219	0.8312	0.8418	0.8216
WBSMDA	0.8163	0.8216	0.8097	0.8277	0.8391	0.8163

Bolded numbers are the best performance in each category.

**FIGURE 3:**
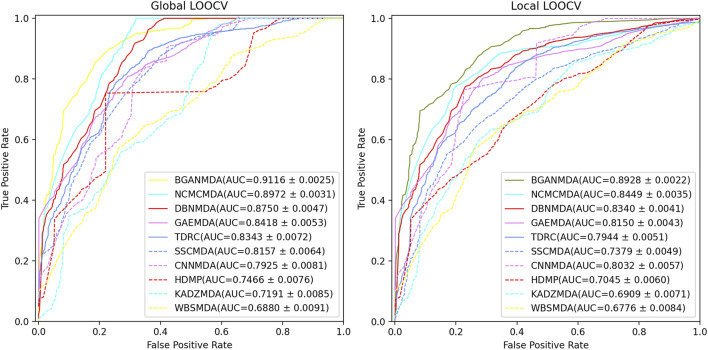
The performance of BGANMDA and the other nine disease-related miRNA prediction methods, namely NCMCMDA, DBNMDA, GAEMDA, TDRC, SSCMDA, CNNMDA, HDMP, KADZMDA and WBSMDA, were compared based on the ROC curve and the AUC value in global and local LOOCV. BGANMDA obtained the AUC and standard deviation of 0.9116 ± 0.0025 in global LOOCV and 0.8928 ± 0.0022 in local LOOCV, respectively.

In LOOCV, the distinction between global and local LOOCV depends on whether the whole miRNA-disease information is included simultaneously specifically, in global LOOCV, the focus was on all underlying pairwise miRNA-disease correlations and each known association between a specific disease and miRNA as test sample was excluded in turn. In contrast, in local LOOCV, only the unknown miRNA-disease associations concerned in test samples were ranked by comparing the association scores. In the rank list, a threshold was given: samples with miRNA-disease association prediction scores above the threshold were considered true positives (TP). For each given threshold, it was possible to find the corresponding TP to determine the true positive ratio (sensitivity). Similarly, the false negatives among the candidate samples could be obtained by setting a threshold, and the corresponding false-positive ratio (1-specificity). The term sensitivity denotes the ratio of the test sample ranking over a specific threshold, whereas specificity indicates the proportion of unknown miRNA-disease association samples ranked under this threshold. Based on the results obtained, the receiver operating characteristic curve was drawn and the AUC at different thresholds was calculated. The higher the AUC value, the better the model performance; if it was close to 1, the BGANMDA was considered an excellent predictor of potential miRNA-disease correlations, whereas the association was regarded as a random prediction if the AUC value was close to 0.5. To comprehensively verify BGANMDA’s predictive ability for complex disease-related miRNAs, we compared it with other nine models for global and local LOOCV. As shown in Figure 3, BGANMDA acquired the AUC and standard deviation of 0.9116 ± 0.0025 in global LOOCV and 0.8928 ± 0.0022 in local LOOCV, respectively. Obviously, our model superior to other advanced methods, which recorded values of 0.8972 ± 0.0031, 0.8750 ± 0.0047, 0.8418 ± 0.0053, 0.8343 ± 0.0072, 0.8157 ± 0.0064, 0.7925 ± 0.0081, 0.7466 ± 0.0076, 0.7191 ± 0.0085, and 0.6880 ± 0.0091 in global LOOCV. However, in local LOOCV, the CNNMDA (0.8032 ± 0.0057) obtained a higher AUC value, while the other methods recorded lower ones, as observed for the NCMCMDA (0.8449 ± 0.0035), DBNMDA (0.8340 ± 0.0041), GAEMDA (0.8150 ± 0.0043), TDRC (0.7944 ± 0.0051), SSCMDA (0.7379 ± 0.0049), HDMP (0.7045 ± 0.0060), KADZMDA (0.6909 ± 0.0071), and WBSMDA (0.6776 ± 0.0084). We assumed that CNNMDA could outperform other methods in local LOOCV because of the effective pattern of convolutional feature extracting. NCMCMDA integrated neighborhood constraint with matrix completion, aiming at transforming the task of recovering the missing miRNA–disease associations into an optimization problem. As can be seen, though NCMCMDA has a good performance in global and local LOOCV, the insufficiency of present known miRNA-disease associations which NCMCMDA strongly depends on will limit the prediction performance. Compared with the other advanced models, BGANMDA has the advantage of excellent learning capability for underlying similar traits, making it inclusive and robust to the lack of unknown miRNA-disease associations.

### 3.2 Case Studies

In order to further evaluate the practical capability of the BGANMDA to predict the potential disease-related miRNAs, three different case studies of fatal cancers were considered, namely the neoplasms of the colon, esophagus, and kidney. Here, the known information on the associations between microRNAs and diseases obtained from the HMDD v 3.2 databases was used as the positive samples for the BGANMDA, and the miRNAs were prioritized based on the predicted score given by the model. Then, the top 50 forecast candidates were ranked based on the dbDEMC database (Yang et al., 2017) and HMDD v3.2 database.

Colon neoplasm is a dangerous malignant tumor causing a high mortality rate in humans, and its morbidity rates are only second to those of esophageal and gastric cancers (Brody, 2015; Ji et al., 2018). Studies have predicted the occurrence of 101,420 new colon neoplasm cases, representing 8.3% of all new cancer cases reported in the United States in 2019, which will result in the death of 51,200 people (Siegel et al., 2019). Thus, it is urgent to develop sensitive and novel biomarkers that can effectively and timely detect colon neoplasms. Studies have confirmed that miRNAs are becoming a crucial target for colon tumor prevention, diagnosis, and therapy. Some research revealed that the abundance of hsa-mir-145 is negatively correlated with its expression in colon neoplasm cells (Wang et al., 2012), a relationship confirmed by luciferase reporter assay. In addition (Yu et al., 2018), identified the association between has-mir-21–5p and the clinicopathological features of colon adenocarcinoma (CODA) patients, as well as its overexpression in CODA cells. The experiments conducted revealed that has-mir-21–5p promoted the migration, and proliferation of colon neoplasm cells, and invasion of tissues, by inhibiting CHL1 expression. It was also shown that the overexpression of has-mir-143 raised oxaliplatin-induced apoptosis relavent to oxygen generation (Gomes et al., 2018). This suggests that has-mir-143 may bypass oxaliplatin resistance in the cells of HCT116 human colon neoplasm by increasing oxidative stress. In this study, the BGANMDA was used to determine the potential miRNAs associated with colon neoplasms and 100, 90, and 92%, were confirmed in the top 10, 20, and 50, respectively, based on the dbDEMC and HMDD v3.2 (see Table 2).

**TABLE 2:** Based on the known associations provided by dbDEMC and HMDD v3.2, the top 50 miRNAs related to colon neoplasm were predicted by employing BGANMDA model, and 46 predictions were confirmed based on dbDEMC and miR2Disease. The first column records the top 1–25 predicted potentially related miRNAs and the third column records the 26–50 predicted potentially relevant miRNAs.

miRNA	Evidence	miRNA	Evidence
hsa-mir-145	dbDEMC; HMDD v3.2	hsa-mir-139–5p	dbDEMC; HMDD v3.2
hsa-mir-21	dbDEMC	hsa-let-7c	dbDEMC; HMDD v3.2
hsa-mir-143	dbDEMC; HMDD v3.2	hsa-mir-96	dbDEMC; HMDD v3.2
hsa-mir-195	dbDEMC; HMDD v3.2	hsa-mir-106a	dbDEMC; HMDDv3.2
hsa-mir-502–5p	dbDEMC	hsa-mir-628–3p	HDMM v3.2
hsa-mir-215	dbDEMC; HMDD v3.2	hsa-mir-210	dbDEMC
hsa-mir-503	dbDEMC	hsa-mir-140–5p	dbDEMC
hsa-mir-100	dbDEMC	hsa-mir-20a	dbDEMC
hsa-mir-155	dbDEMC	hsa-mir-28–5p	HMDD v3.2
hsa-mir-497	dbDEMC; HMDD v3.2	hsa-mir-342–3p	dbDEMC; HMDD v3.2
hsa-mir-548d-3p	dbDEMC; HMDD v3.2	hsa-mir-556–5p	dbDEMC
hsa-mir-150	unconfirmed	hsa-mir-23a	dbDEMC
hsa-mir-552	dbDEMC; HMDD v3.2	hsa-mir-93	dbDEMC; HMDD v3.2
hsa-mir-650	HMDD v3.2	hsa-mir-133b	HMDD v3.2
hsa-mir-491–5p	dbDEMC	hsa-mir-518b	dbDEMC; HMDD v3.2
hsa-mir-183	dbDEMC	hsa-mir-581	unconfirmed
hsa-mir-30a	HMDD v3.2	hsa-mir-421	dbDEMC; HMDD v3.2
hsa-mir-182	dbDEMC; HMDD v3.2	hsa-mir-192a	dbDEMC
hsa-mir-378	HMDD v3.2	hsa-mir-32–3p	dbDEMC
hsa-mir-34a	unconfirmed	hsa-mir-18a	dbDEMC; HMDD v3.2
hsa-mir-17	dbDEMC	hsa-mir-330–3p	dbDEMC; HMDD v3.2
hsa-mir-665	dbDEMC	hsa-mir-203	dbDEMC; HMDD v3.2
hsa-mir-155–3p	dbDEMC; HMDD v3.2	hsa-mir-583	HMDD v3.2
hsa-mir-623	HMDD v3.2	hsa-mir-889	unconfirmed
hsa-mir-486–5p	HMDD v3.2	hsa-mir-10b	dbDEMC; HMDD v3.2

In order to illustrate the capability of the BGANMDA to predict diseases with unconfirmed miRNAs, the esophageal neoplasm was selected as a concrete example, based on the HMDD v 3.2 database. More specifically, the validated disease-related microRNAs of this tumor were omitted and it was considered a new disease. Hence, the model only extracted the associated miRNAs of other diseases and all the miRNA-disease similarity information to train its prediction ability. As a dangerous and high-incidence tumor worldwide, the etiology of esophageal neoplasms is associated with inflammation, chronic nitrosamine stimulation, and content of microelements in edibles (Kollarova et al., 2007). A number of studies have revealed that hsa-mir-133b may be a potential therapeutic target for esophageal squamous cell carcinoma. Its overexpression can inhibit the MAPK/ERK and PI3K/AKT signaling pathways by regulating epidermal growth factor receptors to suppress the proliferation and migration, of esophageal squamous carcinoma, and invasion of tissues cells (Zeng et al., 2019). Thus, detecting the existing miRNA biomarkers is of great importance to discover esophageal neoplasm cases. Some studies indicated that hsa-mir-17–5p is a crucial biomarker to predict the response to neoadjuvant chemoradiation therapy in esophageal adenocarcinoma (EAC), which would help improve patient stratification and serve as a new therapeutic target to boost the efficacy of this therapy in EAC (Lynam-Lennon et al., 2017). After the implementation of BGANMDA resulted in values of 100, 95 and 92% in the top 10, 20, and 50 potential miRNAs related to esophageal neoplasm based on the dbDEMC and HMDD v3.2 databases (Supplementary Table S3).

To evaluate the stability of the model performance using various data sources, all the known kidney neoplasm-related miRNA associations and similarity information (obtained from the HMDD v 2.0 database) database and unverified pairwise miRNA-disease associations were used to train its prediction ability. Then, the prediction scores obtained from the model were verified using the HMDD v 3.2 and dbMDEMC databases, and the literature. Kidney neoplasm, also known as renal cancer, is recognized as one of the top 10 frequent diseases, with over 250,000 unheard cases verified each year. Therefore, finding the association between kidney neoplasm progression and the dysregulation of certain miRNAs can accelerate the prevention, diagnosis, and treatment of renal cancer, and reduce their costs (Shephard et al., 2013). Has-mir-429, the second-highest probability associated with kidney neoplasm predicted by the model, has been reported to be downregulated in contrast-induced acute kidney injury (CI-AKI). In this model, the overexpression of mir-429 reduced apoptosis and increased cell viability by targeting PDCD4 to inhibit the NF-B signaling pathway (Niu et al., 2021). According to previous research, hsa-mir-210 can directly target HIF-1 *α* and inhibit the HIF-1 *α* pathway by participating in the molecular response of hypoxic kidney injury *in vitro*, thus protecting renal tumor cells from hypoxia-induced apoptosis (Liu et al., 2017). In addition, hsa-mir-206 also suppresses kidney neoplasm carcinoma proliferation and epithelial-mesenchymal transformation by inhibiting CDK6 expression (Guo et al., 2020b). After training the stable capability of the BGANMDA, the results showed that the accuracy in the determination of potential miRNAs correlated with kidney neoplasm was 90, 95, and 96% in the top 10, 20, and 50, respectively, based on the dbDEMC and HMDD v3.2 databases, as shown in Table 3.

**TABLE 3:** Based on the validated associations provided by dbDEMC and HMDD v3.2, the top 50 miRNAs related to kidney neoplasm were predicted by employing BGANMDA model, and 48 predictions were confirmed based on dbDEMC and miR2Disease. The first column records the top 1–25 predicted potentially relevant miRNAs and the third column records the 26–50 predicted potentially related miRNAs.

miRNA	Evidence	miRNA	Evidence
hsa-mir-21	dbDEMC; HMDD v3.2	hsa-mir-21–5p	dbDEMC; HMDD v3.2
hsa-mir-429	dbDEMC	hsa-mir-548d-3p	Unconfirmed
hsa-mir-299–5p	dbDEMC	hsa-mir-30c-2-3p	dbDEMC; HMDD v3.2
hsa-mir-200c	dbDEMC	hsa-mir-30a-5p	dbDEMC; HMDDv3.2
hsa-mir-204	dbDEMC; HMDD v3.2	hsa-mir-513c-5p	dbDEMC
hsa-mir-1293	HMDD v3.2	hsa-mir-584–3p	dbDEMC; HMDD v3.2
hsa-mir-184	dbDEMC; HMDD v3.2	hsa-mir-20b	HMDD v3.2
hsa-mir-193a-3p	dbDEMC; HMDD v3.2	hsa-mir-18a	HMDD v3.2
hsa-mir-210	unconfirmed	hsa-mir-144–5p	HMDD v3.2
hsa-mir-211–5p	dbDEMC; HMDD v3.2	hsa-mir-244–5p	HMDD v3.2
hsa-mir-199a-5p	dbDEMC	hsa-mir-106b	dbDEMC; HMDD v3.2
hsa-mir-532–5p	dbDEMC; HMDD v3.2	hsa-mir-133b	dbDEMC; HMDD v3.2
hsa-mir-433–3p	dbDEMC; HMDD v3.2	hsa-mir-483–5p	dbDEMC
hsa-mir-206	HMDD v3.2	hsa-mir-580–5p	dbDEMC; HMDD v3.2
hsa-mir-489–5p	HMDD v3.2	hsa-mir-484	dbDEMC
hsa-mir-660–5p	dbDEMC	hsa-mir-363	dbDEMC
hsa-mir-3654	dbDEMC; HMDD v3.2	hsa-mir-93	dbDEMC; HMDD v3.2
hsa-mir-196a-3p	unconfirmed	hsa-mir-342–3p	dbDEMC; HMDD v3.2
hsa-mir-320b	dbDEMC; HMDD v3.2	hsa-mir-215	dbDEMC; HMDD v3.2
hsa-mir-199b-5p	dbDEMC; HMDD v3.2	hsa-mir-194	dbDEMC
hsa-mir-301b-3p	dbDEMC	hsa-mir-6843–3p	HMDD v3.2
hsa-mir-199b-3p	dbDEMC	hsa-mir-496	HMDD v3.2
hsa-mir-17–3p	dbDEMC; HMDD v3.2	hsa-mir-30e-3p	HMDD v3.2
hsa-mir-199a-3p	HMDD v3.2	hsa-mir-415a	dbDEMC; HMDD v3.2
hsa-mir-676–5p	dbDEMC; HMDD v3.2	hsa-mir-185	dbDEMC; HMDD v3.2

## 4 Discussion and Conclusion

Predicting the underlying associations of miRNA-disease pairs contributes to the understanding of disease mechanisms at the miRNA level, ultimately resulting in better prevention, diagnosis, and treatment. In the present study the model known as BGANMDA, based on auto-encoder and traditional generative adversarial networks, was proposed to determine the probability score of unknown miRNA-disease pairs by constructing the miRNAs (diseases) similarity network and GIP kernel similarity of miRNAs (diseases). The BGANMDA showed a superior performance compared to other advanced methods in three types of cross validation, which also reflected its stable capability. Furthermore, case studies of various diseases also confirmed that the model’s predictions are reliable and accurate.

The model’s successful performance of can be illustrated by the following factors. First, the similarity network was constructed from miRNAs (diseases) as traits to train the model. Second, the BGANMDA retained the advantages of both the auto-encoder and GAN, which can automatically recognize the comprehensive similarity characteristics of miRNAs and diseases, eliminate noise, and reduce dimensions. In addition, the model had an excellent performance in terms of learning the annotated biological patterns. Third, and most importantly, it achieved a bidirectional GAN structure, which means that the model’s encoder mapped the data points *x* into latent space and the generator’s sampling noise from the latent space to generate new miRNA-disease associations. Ultimately, the encoder and generator of BGANMDA can invert each other based on the joint probability distribution.

However, the model has some limitations. First, the parameter values proposed were set as default, so it was not possible to further consider whether the performance would be impacted. Parameter settings play an important role in assisting the model to learn privileged information from the eigenvectors, particularly for complex associated features. Second, the model’s strongly relied on similarity features, which were computed based on handcrafted measurements. Third, the information in each network layer could not be shared and propagated well, because the component of the auto-encoder was used for compressing the features into low dimensions and learning the latent representation.

## Data Availability

The original contributions presented in the study are included in the article/Supplementary Material; further inquiries can be directed to the corresponding authors.
