# Supplementary material for: Application of Bidirectional Generative Adversarial Networks to Predict Potential miRNAs Associated With Diseases
Source: Front Genet. 2022 Jul 12;13:936823. doi: 10.3389/fgene.2022.936823 (PMC9314862; doi:10.3389/fgene.2022.936823)
Supplement: Supplementary file 1 [file DataSheet1.PDF]

Supplementary Material

1 SUPPLEMENTARY TABLES AND FIGURES

Noting that we uploaded 4 figures and 4 tables altogether in this supplementary material.

Table S1. The AUC AUPR, F1-Scores, Recall, Precision and MCC of ten methods on miRNA-disease associations prediction task in five-fold cross validation.

	AUC	AUPR	F1-score	Recall	Precision	MCC
BGANMDA	**0.9316**	**0.9237**	0.9024	0.8968	**0.9215**	**0.8913**
NCMCMDA	0.9187	0.9093	**0.9031**	0.8621	0.9033	0.8541
DBNMDA	0.8994	0.8972	0.8793	0.8517	0.8802	0.8632
GAEMDA	0.8674	0.8625	0.8609	0.8485	0.8756	0.8427
TDRCMDA	0.8713	0.8706	0.8569	**0.8990**	0.8783	0.8409
SSCMDA	0.8531	0.8654	0.8542	0.8501	0.8697	0.8371
CNNMDA	0.8494	0.8583	0.8512	0.8453	0.8724	0.8347
HDMP	0.8226	0.8407	0.8373	0.8486	0.8531	0.8287
KADZMDA	0.8317	0.8351	0.8219	0.8312	0.8418	0.8216
WBSMDA	0.8163	0.8216	0.8097	0.8277	0.8391	0.8163

Note: Bolded numbers are the best performance in each category.

Table S2. Based on the known associations provided by dbDEMC and HMDD v3.2, the top 50 miRNAs related to colon neoplasm were predicted by employing BGANMDA model, and 46 predictions were confirmed based on dbDEMC and miR2Disease. The first column records the top 1–25 predicted potentially related miRNAs and the third column records the 26–50 predicted potentially relevant miRNAs

miRNA	evidence	miRNA	evidence
hsa-mir-145	dbDEMC;HMDD v3.2	hsa-mir-139-5p	dbDEMC;HMDD v3.2
hsa-mir-21	dbDEMC	hsa-let-7c	dbDEMC;HMDD v3.2
hsa-mir-143	dbDEMC;HMDD v3.2	hsa-mir-96	dbDEMC;HMDD v3.2
hsa-mir-195	dbDEMC;HMDD v3.2	hsa-mir-106a	dbDEMC;HMDDv3.2
hsa-mir-502-5p	dbDEMC	hsa-mir-628-3p	HDMM v3.2
hsa-mir-215	dbDEMC;HMDD v3.2	hsa-mir-210	dbDEMC
hsa-mir-503	dbDEMC	hsa-mir-140-5p	dbDEMC
hsa-mir-100	dbDEMC	hsa-mir-20a	dbDEMC
hsa-mir-155	dbDEMC	hsa-mir-28-5p	HMDD v3.2
hsa-mir-497	dbDEMC;HMDD v3.2	hsa-mir-342-3p	dbDEMC;HMDD v3.2
hsa-mir-548d-3p	dbDEMC;HMDD v3.2	hsa-mir-556-5p	dbDEMC
hsa-mir-150	unconfirmed	hsa-mir-23a	dbDEMC
hsa-mir-552	dbDEMC;HMDD v3.2	hsa-mir-93	dbDEMC;HMDD v3.2
hsa-mir-650	HMDD v3.2	hsa-mir-133b	HMDD v3.2
hsa-mir-491-5p	dbDEMC;	hsa-mir-518b	dbDEMC;HMDD v3.2
hsa-mir-183	dbDEMC	hsa-mir-581	unconfirmed
hsa-mir-30a	HMDD v3.2	hsa-mir-421	dbDEMC;HMDD v3.2
hsa-mir-182	dbDEMC;HMDD v3.2	hsa-mir-192a	dbDEMC
hsa-mir-378	HMDD v3.2	hsa-mir-32-3p	dbDEMC;
hsa-mir-34a	unconfirmed	hsa-mir-18a	dbDEMC;HMDD v3.2
hsa-mir-17	dbDEMC	hsa-mir-330-3p	dbDEMC;HMDD v3.2
hsa-mir-665	dbDEMC	hsa-mir-203	dbDEMC;HMDD v3.2
hsa-mir-155-3p	dbDEMC;HMDD v3.2	hsa-mir-583	HMDD v3.2
hsa-mir-623	HMDD v3.2	hsa-mir-889	unconfirmed
hsa-mir-486-5p	HMDD v3.2	hsa-mir-10b	dbDEMC;HMDD v3.2

Table S3. Based on the experimental associations provided by dbDEMC and HMDD v3.2, the top 50 miRNAs related to esophageal neoplasm were predicted by employing BGANMDA model, and 46 predictions were confirmed based on dbDEMC and miR2Disease. The first column records the top 1–25 predicted potentially relevant miRNAs and the third column records the 26–50 predicted potentially related miRNAs

miRNA	evidence	miRNA	evidence
hsa-mir-21	HMDD v3.2	hsa-mir-92a-3p	dbDEMC;HMDD v3.2
hsa-mir-4739	dbDEMC;HMDD v3.2	hsa-mir-19b-3p	HMDD v3.2
hsa-mir-320e	dbDEMC;HMDD v3.2	hsa-mir-3192-3p	dbDEMC;HMDD v3.2
hsa-mir-508-5p	HMDD v3.2	hsa-mir-606	dbDEMC;HMDDv3.2
hsa-mir-133b	dbDEMC;HMDD v3.2	hsa-mir-4734	dbDEMC
hsa-mir-103a-3p	dbDEMC	hsa-mir-877-5p	dbDEMC
hsa-mir-17-5p	dbDEMC	hsa-mir-365b-5p	dbDEMC;HMDD v3.2
hsa-mir-30a-3p	dbDEMC;HMDD v3.2	hsa-mir-149-3p	HMDD v3.2
hsa-mir-518c-3p	dbDEMC;HMDD v3.2	hsa-mir-375	dbDEMC
hsa-mir-125b-1-3p	dbDEMC;HMDD v3.2	hsa-mir-143	dbDEMC;HMDD v3.2
hsa-mir-4741	dbDEMC;HMDD v3.2	hsa-mir-203	dbDEMC;HMDD v3.2
hsa-mir-485-3p	dbDEMC;HMDD v3.2	hsa-mir-145	dbDEMC
hsa-mir-4685-3p	HMDD v3.2	hsa-mir-483-205	dbDEMC;HMDD v3.2
hsa-mir-30a-5p	HMDD v3.2	hsa-mir-31	dbDEMC;HMDD v3.2
hsa-mir-603	dbDEMC;HMDD v3.2	hsa-mir-141	HMDD v3.2
hsa-mir-423-3p	dbDEMC	hsa-mir-34a	HMDD v3.2
hsa-mir-139-3p	unconfirmed	hsa-mir-100	HMDD v3.2
hsa-mir-15b-5p	dbDEMC;HMDD v3.2	hsa-mir-25	dbDEMC;HMDD v3.2
hsa-mir-1224-5p	dbDEMC	hsa-mir-486	unconfirmed
hsa-mir-564	dbDEMC	hsa-mir-155	dbDEMC;HMDD v3.2
hsa-mir-130b-3p	HMDD v3.2	hsa-mir-22	dbDEMC;HMDD v3.2
hsa-mir-26a-5p	dbDEMC;HMDD v3.2	hsa-mir-10b	unconfirmed
hsa-mir-5193	dbDEMC;HMDD v3.2	hsa-mir-192	deDEMC;HMDD v3.2
hsa-mir-628-5p	unconfirmed	hsa-mir-200a	HMDD v3.2
hsa-mir-345-3p	dbDEMC;HMDD v3.2	hsa-mir-99b	HMDD v3.2

Table S4. Based on the validated associations provided by dbDEMC and HMDD v3.2, the top 50 miRNAs related to kidney neoplasm were predicted by employing BGANMDA model, and 48 predictions were confirmed based on dbDEMC and miR2Disease. The first column records the top 1–25 predicted potentially relevant miRNAs and the third column records the 26–50 predicted potentially related miRNAs

miRNA	evidence	miRNA	evidence
hsa-mir-21	dbDEMC;HMDD v3.2	hsa-mir-21-5p	dbDEMC;HMDD v3.2
hsa-mir-429	dbDEMC	hsa-mir-548d-3p	unconfirmed
hsa-mir-299-5p	dbDEMC	hsa-mir-30c-2-3p	dbDEMC;HMDD v3.2
hsa-mir-200c	dbDEMC	hsa-mir-30a-5p	dbDEMC;HMDDv3.2
hsa-mir-204	dbDEMC;HMDD v3.2	hsa-mir-513c-5p	dbDEMC
hsa-mir-1293	HMDD v3.2	hsa-mir-584-3p	dbDEMC;HMDD v3.2
hsa-mir-184	dbDEMC;HMDD v3.2	hsa-mir-20b	HMDD v3.2
hsa-mir-193a-3p	dbDEMC;HMDD v3.2	hsa-mir-18a	HMDD v3.2
hsa-mir-210	unconfirmed	hsa-mir-144-5p	HMDD v3.2
hsa-mir-211-5p	dbDEMC;HMDD v3.2	hsa-mir-244-5p	HMDD v3.2
hsa-mir-199a-5p	dbDEMC	hsa-mir-106b	dbDEMC;HMDD v3.2
hsa-mir-532-5p	dbDEMC;HMDD v3.2	hsa-mir-133b	dbDEMC;HMDD v3.2
hsa-mir-433-3p	dbDEMC;HMDD v3.2	hsa-mir-483-5p	dbDEMC
hsa-mir-206	HMDD v3.2	hsa-mir-580-5p	dbDEMC;HMDD v3.2
hsa-mir-489-5p	HMDD v3.2	hsa-mir-484	dbDEMC
hsa-mir-660-5p	dbDEMC	hsa-mir-363	dbDEMC
hsa-mir-3654	dbDEMC;HMDD v3.2	hsa-mir-93	dbDEMC;HMDD v3.2
hsa-mir-196a-3p	unconfirmed	hsa-mir-342-3p	dbDEMC;HMDD v3.2
hsa-mir-320b	dbDEMC;HMDD v3.2	hsa-mir-215	dbDEMC;HMDD v3.2
hsa-mir-199b-5p	dbDEMC;HMDD v3.2	hsa-mir-194	dbDEMC
hsa-mir-301b-3p	dbDEMC	hsa-mir-6843-3p	HMDD v3.2
hsa-mir-199b-3p	dbDEMC	hsa-mir-496	HMDD v3.2
hsa-mir-17-3p	dbDEMC;HMDD v3.2	hsa-mir-30e-3p	HMDD v3.2
hsa-mir-199a-3p	HMDD v3.2	hsa-mir-415a	dbDEMC;HMDD v3.2
hsa-mir-676-5p	dbDEMC;HMDD v3.2	hsa-mir-185	dbDEMC;HMDD v3.2

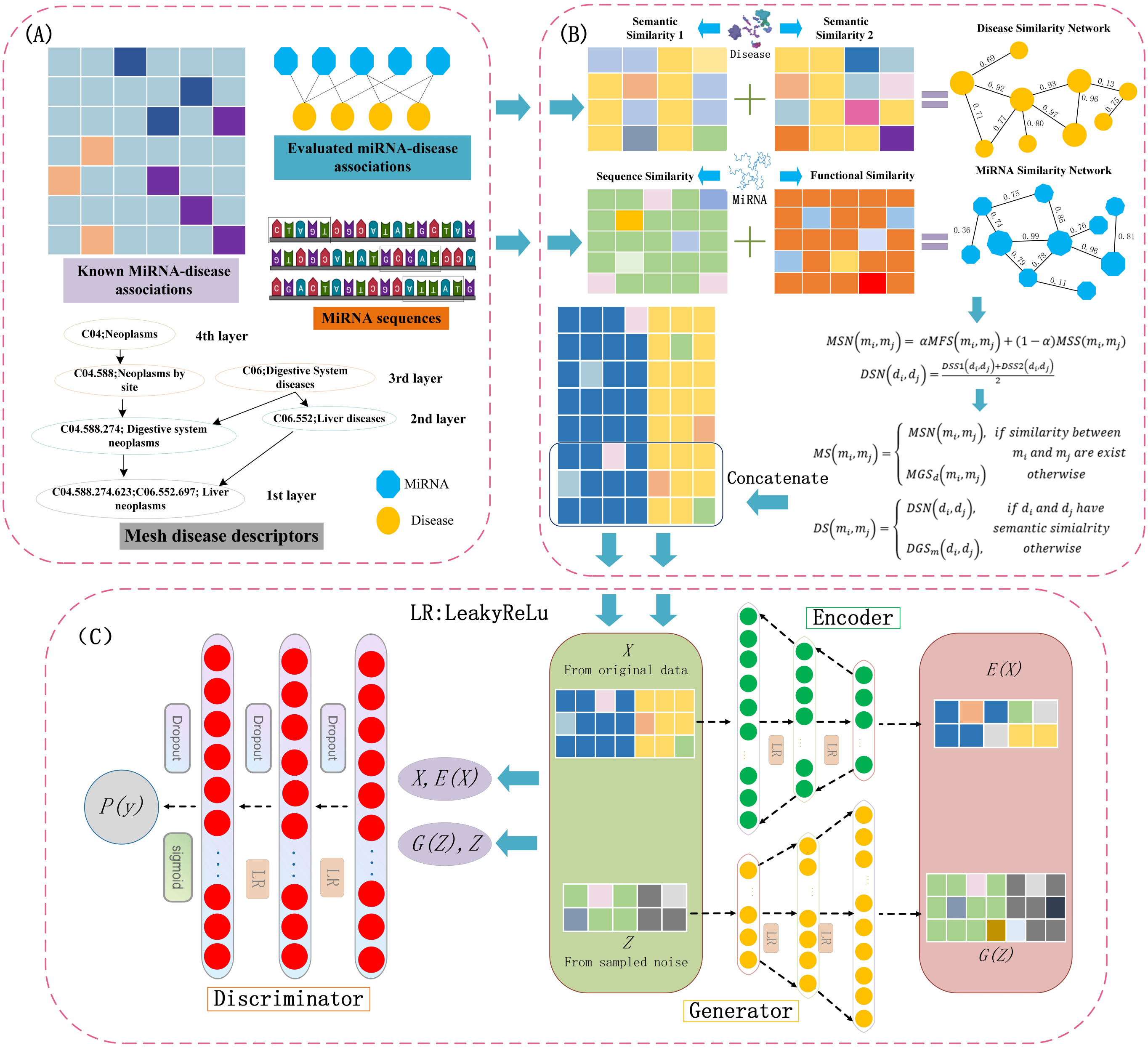

Figure S1. Flowchart of potential miRNA-disease association prediction based on the BGANMDA model, **(A)** is the multi-source information of miRNAs and diseases, **(B)** is the details of constructing both miRNAs and diseases similarity network, and **(C)** is the structure of BGANMDA.

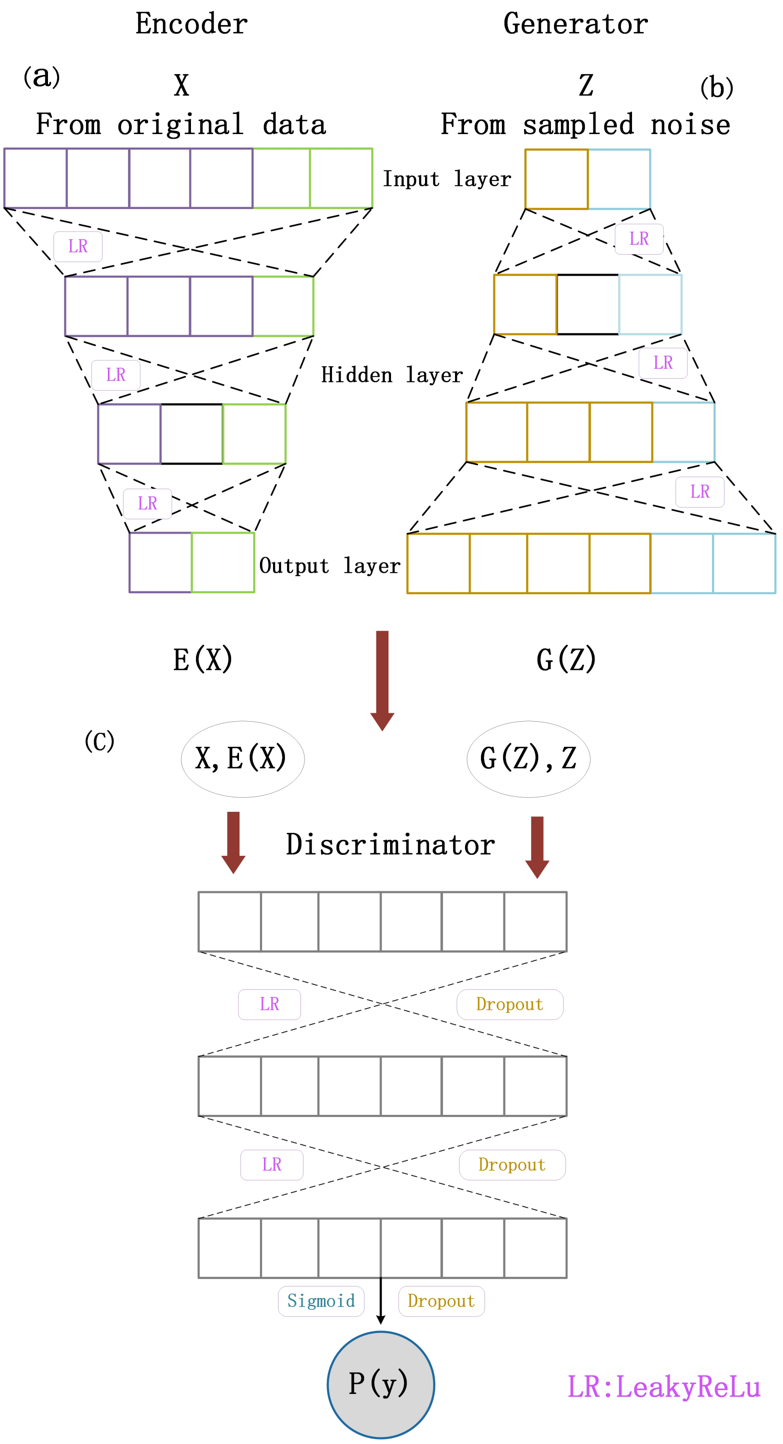

Figure S2. The structure of bidirectional generative adversarial network. **(a)**.Encoder contains three network layers, which takes *X* (from original data) as input, and the dimension of each layer reduced gradually. **(b)**. Generator has three network layers and which dimension increases layer by layer, taking sampled noise as input. **(c)**. Discriminator feeds the data pairs (*X,E(X)*) or (*G(Z),Z*) into input layer and employs sigmoid function to output the predicted results.

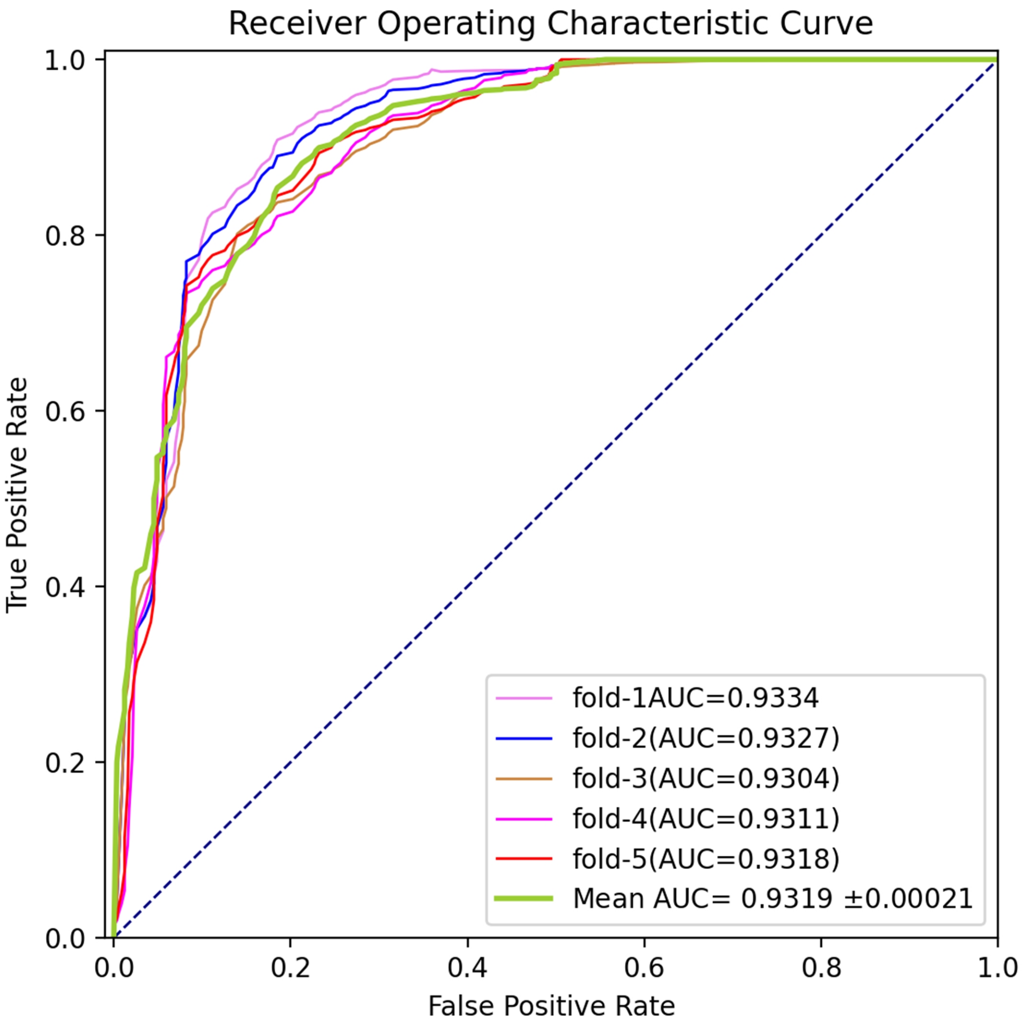

Figure S3. ROC curves performed in 5-flod cross validation by BGANMDA, which obtained the mean AUC value and standard deviation of 0.9319 ± 0.0021.

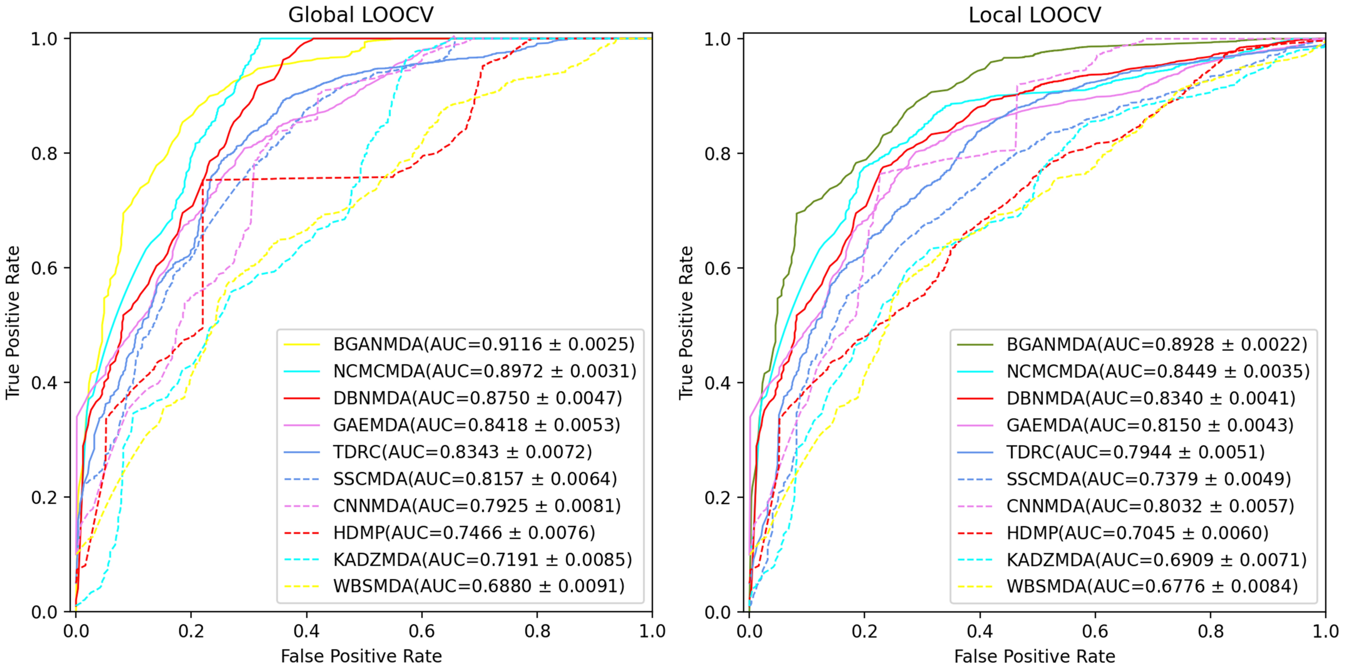

Figure S4. The performance of BGANMDA and the other nine disease-related miRNA prediction methods,namely NCMCMDA, DBNMDA, GAEMDA, TDRC, SSCMDA, CNNMDA, HDMP, KADZMDA and WBSMDA, were compared based on the ROC curve and the AUC value in global and local LOOCV. BGANMDA obtained the AUC and standard deviation of 0.9116 ± 0.0025 in global LOOCV and 0.8928 ± 0.0022 in local LOOCV, respectively.
